# A yeast two-hybrid system for the screening and characterization of small-molecule inhibitors of protein–protein interactions identifies a novel putative Mdm2-binding site in p53

**DOI:** 10.1186/s12915-017-0446-7

**Published:** 2017-11-09

**Authors:** Jin Huei Wong, Mohammad Alfatah, Mei Fang Sin, Hong May Sim, Chandra S. Verma, David P. Lane, Prakash Arumugam

**Affiliations:** 1 Bioinformatics Institute, 30 Biopolis Street, #07-01, Matrix, Singapore, 138671 Singapore; 2 Department of Pharmacy, National University of Singapore, National University of Singapore 18 Science Drive 4, Singapore, 117543 Singapore; 3 Department of Biological Sciences, National University of Singapore, 14 Science Drive, Singapore, 117543 Singapore; 4 Nanyang Technological University, School of Biological Sciences, 50 Nanyang Drive, Singapore, 637551 Singapore; 5 The p53 Laboratory, 8A Biomedical Grove, Singapore, 138648 Singapore

**Keywords:** Yeast two-hybrid assay, Protein–protein interaction inhibitors, p53-Mdm2 interaction

## Abstract

**Background:**

Protein–protein interactions (PPIs) are fundamental to the growth and survival of cells and serve as excellent targets to develop inhibitors of biological processes such as host-pathogen interactions and cancer cell proliferation. However, isolation of PPI inhibitors is extremely challenging. While several in vitro assays to screen for PPI inhibitors are available, they are often expensive, cumbersome, and require large amounts of purified protein. In contrast, limited in vivo assays are available to screen for small-molecule inhibitors of PPI.

**Methods:**

We have engineered a yeast strain that is suitable for screening of small-molecule inhibitors of protein-protein interaction using the Yeast 2-hybrid Assay. We have optimised and validated the assay using inhibitors of the p53-Mdm2 interaction and identified a hitherto unreported putative Mdm2-binding domain in p53.

**Results:**

We report a significantly improved and thoroughly validated yeast two-hybrid (Y2H) assay that can be used in a high throughput manner to screen for small-molecule PPI inhibitors. Using the p53-Mdm2 interaction to optimize the assay, we show that the p53-Mdm2 inhibitor nutlin-3 is a substrate for the yeast ATP-binding cassette (ABC) transporter Pdr5. By deleting nine ABC transporter-related genes, we generated a ABC9Δ yeast strain that is highly permeable to small molecules. In the ABC9Δ strain, p53-Mdm2 interaction inhibitors, like AMG232 and MI-773, completely inhibited the p53-Mdm2 interaction at nanomolar concentrations in the Y2H assay. In addition, we identified a conserved segment in the core DNA-binding domain of p53 that facilitates stable interaction with Mdm2 in yeast cells and *in vitro*.

**Conclusion:**

The Y2H assay can be utilized for high-throughput screening of small-molecule inhibitors of PPIs and to identify domains that stabilize PPIs.

**Electronic supplementary material:**

The online version of this article (doi:10.1186/s12915-017-0446-7) contains supplementary material, which is available to authorized users.

## Background

A multitude of biological processes are dependent on protein–protein interactions (PPIs). It has been estimated that approximately 350,000 types of PPIs occur in a human cell [1]. This offers innumerable opportunities to develop PPI inhibitors and thus to specifically control cellular processes. However, obtaining a small-molecule inhibitor of PPIs is not trivial. Generally, the interfaces involved in PPIs are flat and large, thereby reducing the probabilities of effective competition by a small-molecule inhibitor [2]. However, there has been some success over the last couple of decades in obtaining small-molecule PPI inhibitors [3]. It has been observed that a few key residues in the PPI interface contribute to the bulk of the binding energy in a PPI [4] and, thus, could be targeted for inhibition by a small molecule.

In order to sample the entire conformational space for PPIs, a simple inexpensive screening assay usable in a high-throughput format is extremely desirable. Although there are in vitro assays involving methods such as isothermal titration calorimetry, surface plasmon resonance, microscale thermophoresis, enzyme-linked immunosorbent assay, fluorescence polarization, fluorescence resonance energy transfer, and the bead-based AlphaScreen assay (Perkin Elmer), they are often labour intensive, expensive, and require large amounts of purified protein [5]. Moreover, in vitro assays may identify compounds that are good PPI inhibitors but may have toxic effects or low cell permeability. Therefore, it is advantageous to have an economic, high throughput in vivo assay to screen for PPI inhibitors.

**Fig. 1:**
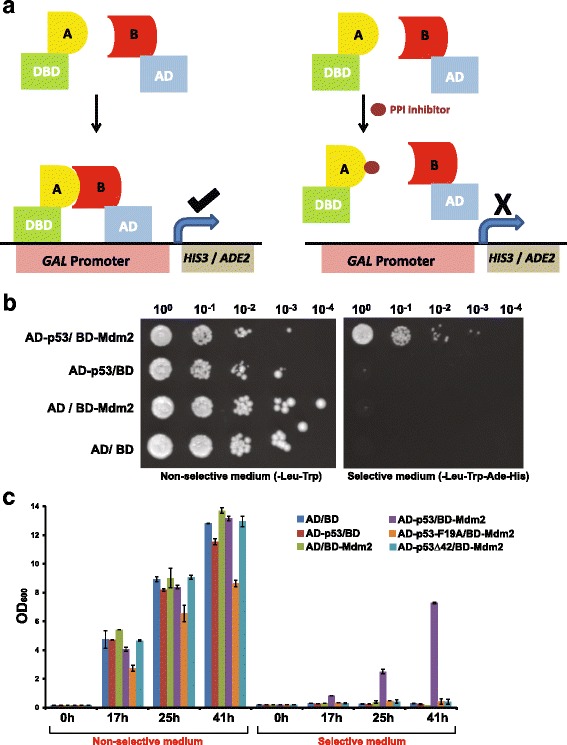
p53 interacts with Mdm2 in the yeast two-hybrid (Y2H) assay. **a** Schematic showing the use of the Y2H assay in identifying interacting proteins (left panel) and inhibitors of protein–protein interactions (right panel). **b** Log-phase cultures of AH109 yeast cells containing plasmids encoding either Gal4 AD-p53/Gal4 BD-Mdm2, Gal4 AD-p53/Gal4 BD, Gal4 AD/Gal4 BD-Mdm2, or Gal4 AD/Gal4 BD were washed in water and plated at different dilutions on non-selective (-Leu-Trp) and selective (-Leu-Trp-Ade-His) plates and incubated at 30 °C for 3 days. **c** Overnight cultures of AH109 yeast cells containing plasmids encoding either Gal4 AD-p53/Gal4 BD-Mdm2, Gal4 AD-p53/Gal4 BD, Gal4 AD/Gal4 BD-Mdm2, Gal4 AD/Gal4 BD, Gal4 AD-p53-F19A/Gal4 BD-Mdm2, or Gal4 AD-p53(Δ42)/Gal4 BD-Mdm2 in non-selective medium were washed in water and inoculated into selective and non-selective medium at OD_600_ = 0.2 in duplicates. For each strain, growth as measured by average OD_600_ of duplicate cultures is plotted against time. Ends of the vertical bar indicate the OD_600_ values of the duplicate cultures

The yeast two-hybrid (Y2H) assay is a powerful tool to identify binary PPIs [6] by exploiting the modular nature of the yeast Gal4 transcription factor. In this assay, the DNA-binding domain and activation domain of Gal4 are fused to two proteins of interest. If the two proteins of interest physically interact, an active Gal4 transcription factor is generated, thereby driving expression of reporter genes under the control of the *GAL* promoter (Fig. 1a, left panel). Apart from confirming an interaction between two proteins, this assay has been pivotal in discovering novel binding proteins. The Y2H assay has been used in developing binary protein interactome maps in model organisms such as yeast [7] and humans [8].

The Y2H assay can also be used to identify domains and amino acid residues required for PPIs. Deletion or replacement of amino acid residues critical for PPI or treatment with small-molecule PPI inhibitors will result in loss of reporter gene activity (Fig. 1a, right panel). It is possible to have a positive selection for screening of mutations or compounds that affect PPIs. For example, by placing the *URA3* gene under the *GAL* promoter, one could screen for mutations or PPI inhibitors that rescue the lethality of yeast cells grown on medium containing 5-fluoroorotic acid; this approach is referred to as the reverse Y2H assay and was proposed 20 years ago [9, 10]. However, there are very few reports of its use in screening of PPI inhibitors [11, 12]. It has been acknowledged that low permeability of yeast cells to small molecules could limit the use of Y2H methods to screen for PPI inhibitors [13].

To explore the use of the Y2H assay to screen for inhibitors of PPIs, we chose the p53-Mdm2 interaction, for which there are several small-molecule inhibitors available. p53 is a master transcription factor that plays a key role in the regulation of cell cycle arrest, DNA damage response, senescence, and apoptosis [14]; it is mutated in more than 50% of cancers [15]. p53 is inhibited by Mdm2, a ubiquitin ligase that is often overexpressed in tumors [16]. By binding to the N-terminal transactivation domain of p53, Mdm2 inhibits its transcriptional activity, ubiquitinates and targets it for proteosomal degradation, and excludes it from the nucleus. Inhibition of the p53-Mdm2 interaction leads to activation of p53 and an increase in its tumor suppressive ability. The p53-Mdm2 interaction can be attributed to three key hotspot residues (Phe19, Trp23, and Leu26) in p53 that bind to a hydrophobic pocket on the surface of the Mdm2’s N-terminal domain [17] (Additional file 1: Figure S1A). Small-molecule inhibitors, such as nutlin, AMG232, and MI-773, bind to the hydrophobic pocket of Mdm2 and inhibit the p53-Mdm2 interaction by mimicking the interaction of the three hydrophobic residues [18–21] (Additional file 1: Figure S1B–D). Binding of Mdm2 to full-length p53 was observed to be approximately 10-fold stronger than the N-terminal domain of p53 (amino acid residues 1–93) [22], indicating the presence of additional domains in p53 that interact with Mdm2. Two such domains have thus far been reported; the DNA-binding domain of p53 (residues 234–286 within the conserved Boxes IV and V) has been shown to interact with the central acidic domain of Mdm2 [23] and the carboxyl-terminal regulatory domain of p53 (residues 367–393) interacts with the N-terminal domain of Mdm2 (residues 10–139) [24].

In this paper, we report on an improved version of the Y2H assay that can be used to screen for inhibitors of PPI in a high throughput manner. By deleting nine genes involved in ABC transporter gene function and expression, we constructed a yeast strain that is highly permeable to small molecules. We show that nutlin, AMG232, and MI-773 can inhibit the p53-Mdm2 interaction in the Y2H assay. We demonstrate that nutlin is a substrate for the yeast ABC transporter Pdr5. In addition, we suggest that the Box II region in the core DNA-binding domain of p53 interacts with the N-terminal domain of Mdm2 and makes the p53-Mdm2 interaction recalcitrant to small-molecule inhibition in the Y2H assay.

## Results

### p53 interacts with Mdm2 in the Y2H assay

The p53-Mdm2 interaction is of immense biomedical importance and is a focus of several screens as a target for small-molecule inhibitors [18]. As there are many small-molecule inhibitors available, it is an ideal system to optimize the Y2H assay [18]. We first established a Y2H assay to detect the p53-Mdm2 interaction. We fused the full-length human p53 (Hp53) to the Gal4 activation domain (Gal4 AD) and the full-length human Mdm2 (hMdm2) to the Gal4 DNA-binding domain (Gal4 BD), and introduced plasmids encoding them into the Y2H reporter strain AH109. AH109 has *HIS3* and *ADE2* genes under the *GAL4* promoter and an interaction between Gal4 AD- and Gal4 BD-fusion proteins results in growth of yeast cells in Synthetic Dropout (SD) medium lacking histidine and adenine (Fig. 1a, left panel). As expected, only cells containing both plasmids encoding Gal4 AD-p53 and Gal4 DBD-Mdm2, but not the control cells, were able to grow in SD medium lacking histidine and adenine (Fig. 1b). Either deletion of the transactivation domain (1–42) or replacement of the hydrophobic triad residue phenylalanine F19 (which is involved in binding to the hydrophobic pocket of Mdm2) with alanine completely abolished growth of Gal4 AD-p53/Gal4 BD-Mdm2-expressing cells in selective medium, indicating the specificity of the interaction (Fig. 1c).

### Nutlin does not inhibit the p53-Mdm2 interaction in wild-type and *pdr1Δ pdr3Δ pdr5Δ* yeast cells in the Y2H assay

Nutlin is a cis-imidazoline analogue that binds to the p53-binding site in Mdm2 (Additional file 1: Figure S1) and inhibits the p53-Mdm2 interaction [19]. Thus, we tested whether nutlin inhibits the p53-Mdm2 interaction in the Y2H assay. Surprisingly, nutlin had no effect on the growth of p53-Mdm2-expressing cells in selective medium (Additional file 2: Figure S2A). We considered the possibility that nutlin was being expelled from yeast cells by ABC transporter-mediated efflux. We deleted three genes, *PDR1*, *PDR3*, and *PDR5*, which have been reported to restrict permeability of yeast cells to small molecules [25], to construct the *pdr1Δ pdr3Δ pdr5Δ* strain (referred to as ABC3Δ for the remainder of this paper). Pdr1 and Pdr3 are transcription factors for the pleiotropic drug response and Pdr5 is an ABC transporter. In the ABC3Δ strain, nutlin inhibited p53-Mdm2 cell growth at 200 μM. However, nutlin also inhibited the growth of control cells expressing Csm1/Dsn1, a pair of interacting yeast kinetochore proteins [23, 26], indicating that the growth inhibition of yeast cells by nutlin was not specific (Additional file 2: Figure S2B).

### Nutlin inhibits the interaction of p53 (1–52) with Mdm2 in *pdr1Δ pdr3Δ pdr5Δ* cells

**Fig. 2:**
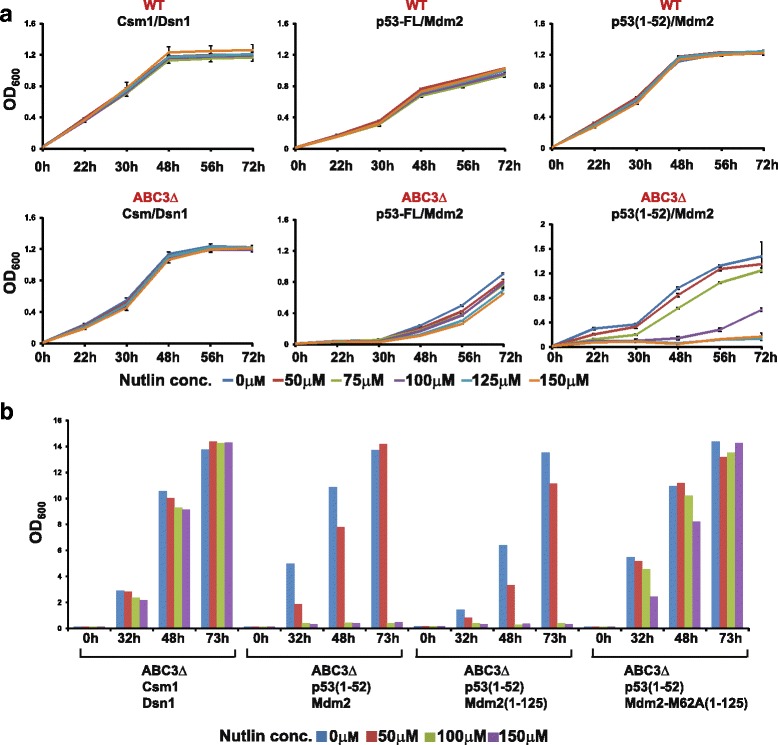
Nutlin inhibits the interaction of p53 (1–52) with Mdm2 in ABC3Δ cells. **a** Overnight cultures of wild-type or ABC3Δ cells containing plasmids encoding either Gal4 AD-Csm1/Gal4 BD-Dsn1 (used as a negative control), Gal4 AD-p53/Gal4 BD-Mdm2, or Gal4 AD-p53 (1–52)/Gal4 BD-Mdm2 in non-selective medium were washed in water and inoculated at OD_600_ = 0.2 into selective medium containing DMSO or nutlin at the indicated concentrations in duplicate. For each strain, growth as measured by average OD_600_ of duplicate cultures is plotted against time. Ends of the vertical bar indicate the OD_600_ values of the duplicate cultures. **b** Overnight cultures of ABC3Δ cells containing plasmids encoding either Gal4 AD-Csm1/Gal4 BD-Dsn1, Gal4 AD-p53 (1–52)/Gal4 BD-Mdm2, Gal4 AD-p53 (1–52)/Gal4 BD-Mdm2 (1–125), or Gal4 AD-p53 (1–52)/Gal4 BD-Mdm2 (1–125)-M62A in non-selective medium were washed in water and inoculated at OD_600_ = 0.2 into selective medium containing DMSO or nutlin at the indicated concentrations. Growth of the cultures was monitored by recording the absorbance at 600 nm at the indicated time points. Data from a repeat experiment are depicted in Additional file 3: Figure S3

We then considered the nature of the p53-Mdm2 interaction. While the transactivation domain of p53 interacts with the N-terminal domain of Mdm2, the core domain and C-terminal regulatory domain of p53 also respectively interact with the acidic- and N-terminal domains of Mdm2 [23, 24]. To eliminate the possible effect of the core domain and C-terminal tail, we expressed p53 (1–52) fused to Gal4 AD and examined the effect of nutlin on its interaction with Gal4 DBD-Mdm2 in ABC3Δ cells. The interaction of p53 (1–52) with Mdm2 was completely inhibited at 125 μM nutlin (Fig. 2a). In contrast, the interaction of full-length p53 with Mdm2 was only partially inhibited (29%), even at 150 μM nutlin. Cells expressing Csm1/Dsn1 in selective medium were unaffected by the addition of 150 μM nutlin, indicating that the inhibition of PPIs by nutlin was specific (Fig. 2a). Moreover, nutlin did not affect the growth of cells expressing p53 (1–52) and Mdm2 in non-selective medium (Additional file 2: Figure S2C). Nutlin did not inhibit the growth of wild-type cells expressing p53 (1–52) and Mdm2, confirming that ABC transporters drive nutlin out of yeast cells (Fig. 2a).

As expected, the interaction of p53 (1–52) with the N-terminal domain of Mdm2 (1–125) was also sensitive to nutlin (Fig. 2b and Additional file 3: Figure S3). To confirm that nutlin inhibits the growth of p53 (1–52)/Mdm2 cells by binding to the N-terminal domain of Mdm2, we introduced the M62A mutation in Mdm2 (1–125), which blocks its interaction with nutlin but retains its interaction with p53 [27]. Crucially, nutlin had no effect on the growth of cells expressing p53 (1–52)/Mdm2 (1–125)-M62A (Fig. 2b and Additional file 3: Figure S3), confirming that nutlin blocks the p53-Mdm2 interaction by binding to Mdm2.

### Nutlin is a substrate for the ABC transporter Pdr5

Inhibition of the p53 (1–52)/Mdm2 interaction by nutlin in ABC3Δ cells but not in wild-type cells (Fig. 2a) suggested that Pdr1, Pdr3, and Pdr5 proteins, either singly or in combination, mediate nutlin efflux from yeast cells. To test which of Pdr1, Pdr3, and Pdr5 proteins contributes to nutlin efflux, we tested the effect of nutlin on the p53 (1–52)/Mdm2 interaction in single mutants. Interestingly, nutlin inhibited the p53 (1–52)/Mdm2 interaction only in *pdr5Δ* cells but not in *pdr1Δ* and *pdr3Δ* cells (Additional file 4: Figure S4), indicating that Pdr5 causes efflux of nutlin from yeast cells.

**Fig. 3:**
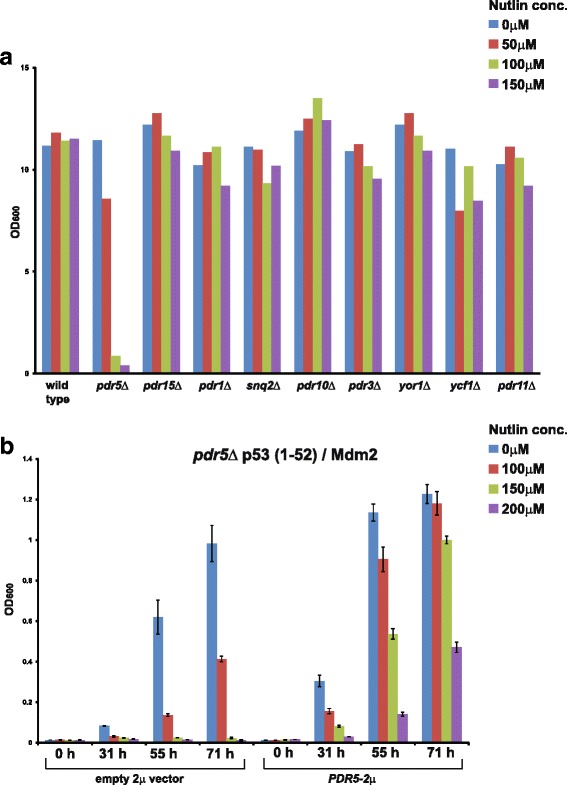
Nutlin is a substrate for the ABC transporter Pdr5. **a** Overnight cultures of either wild-type strain or the indicated deletion strains (*pdr5Δ*, *pdr15Δ*, *pdr1Δ*, etc.) containing plasmids encoding Gal4 AD-p53 (1–52)/Gal4 BD-Mdm2 were washed in water and inoculated at OD_600_ = 0.2 into selective medium containing DMSO or nutlin at the indicated concentrations. Growth of the cultures was monitored by recording the absorbance at 600 nm after 0, 24, 44, and 68 h following inoculation. Growth at 68 h is indicated in the plot. Additional file 5: Figure S5A contains the complete dataset and Additional file 6: Figure S6 contains data from a repeat experiment. **b** Overnight cultures of *pdr5Δ* cells containing plasmids encoding Gal4 AD-p53 (1–52)/Gal4 BD-Mdm2 and Pdr5 or Gal4 AD-p53 (1–52)/Gal4 BD-Mdm2 alone were washed in water and inoculated at OD_600_ = 0.2 into selective medium containing DMSO or nutlin at the indicated concentrations in duplicate. For both strains, growth as measured by average OD_600_ of duplicate cultures is plotted against time. Ends of the vertical bar indicate the OD_600_ values of the duplicate cultures

The *Saccharomyces cerevisiae* proteome is predicted to have 22 ABC transporters [28]. Although only a few of these transporters have been extensively studied, they are known to exhibit broad specificity [28]. To evaluate the specificity of Pdr5 in nutlin efflux, we deleted six genes encoding ABC transporters, namely *PDR15*, *PDR11*, *PDR10*, *YCF1*, *SNQ2*, and *YOR1*, and tested the effect of nutlin on the p53-Mdm2 interaction in the mutants. Strikingly, the p53-Mdm2 interaction was sensitive to nutlin only in *pdr5Δ* cells but not in the other eight mutants (Fig. 3a, Additional file 5: Figure S5A and Additional file 6: Figure S6). For an unrelated drug, rapamycin, *pdr11Δ* and *yor1Δ* were most sensitive (Additional file 5: Figure S5B), confirming that ABC transporters have distinct substrate specificities. Nutlin sensitivity of *pdr5Δ* cells expressing p53 (1–52)/Mdm2 was rescued by introduction of a 2-μ plasmid encoding Pdr5 under the *ADH1* promoter (Fig. 3b). These results indicate that nutlin is a substrate of Pdr5.

**Fig. 4:**
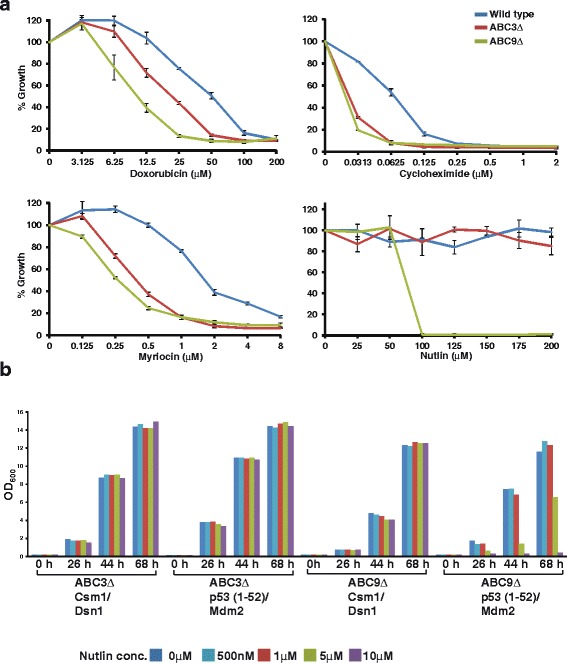
The ABC9Δ strain has increased permeability to several compounds. **a** Overnight cultures of either wild-type, ABC3Δ (*pdr1Δ pdr3Δ pdr5Δ*), or ABC9Δ (*pdr1Δ pdr3Δ pdr5Δ snq2Δ pdr10Δ pdr11Δ pdr15Δ yor1Δ ycf1Δ*) cells in rich medium (YEPD) were washed in water and inoculated at OD_600_ = 0.0625 into YEPD containing either DMSO, doxorubicin, cycloheximide, myriocin, or nutlin at the indicated concentrations in duplicate. For each strain, growth as measured by average OD_600_ of duplicate cultures after 40 h following inoculation was recorded. Percentage inhibition at different concentrations was calculated by normalizing with respect to the growth in DMSO-treated cultures. Ends of the vertical bar indicate values of percentage inhibition for the duplicate cultures. **b** Overnight cultures of ABC3Δ or ABC9Δ cells containing plasmids encoding either Gal4 AD-Csm1/Gal4 BD-Dsn1 or Gal4 AD-p53 (1–52)/Gal4 BD-Mdm2 in non-selective medium were washed in water and inoculated into selective medium at OD_600_ = 0.0625 Growth of the cultures was monitored by recording the absorbance at 600 nm after 0, 26, 44, and 68 h following inoculation. Data from a repeat experiment are shown in Additional file 7: Figure S7

To use the Y2H assay for screening of PPI inhibitors, it is desirable to have a yeast strain that is permeable to a diverse collection of compounds. As ABC transporters have differing substrate specificities, we constructed a strain that lacks several ABC transporter genes. A previous study showed that a yeast strain lacking seven ABC transporter genes (*PDR5*, *PDR15*, *PDR11*, *PDR10*, *YCF1*, *SNQ2*, and *YOR1*) and one gene encoding a transcription factor for ABC transporters (*PDR3*) was viable [29]. We generated the ABC9Δ strain, a derivative of the Y2H strain AH109, which contains the abovementioned eight gene deletions together with deletion of *PDR1*. The ABC9Δ strain was hypersensitive to doxorubicin, myriocin, cycloheximide, and nutlin in comparison to the wild-type strain, indicating that it is permeable to several compounds (Fig. 4a). This is consistent with earlier studies showing that ABC transporter mutants in yeast are sensitive to doxorubicin, myriocin, and cycloheximide [30, 31]. In the ABC9Δ strain, the p53 (1–52)/Mdm2 interaction was sensitive to nutlin at 10 μM in comparison to 100 μM in the ABC3Δ strain (Figs. 4b and Additional file 7: Figure S7). This value (10 μM) fits closely with the potency of nutlin in mammalian cells, indicating that the ABC9Δ strain is an excellent system for small-molecule PPI inhibitor screens [32, 33].

### AMG232 and MI-773 inhibit the p53 (1–52)/Mdm2 interaction in the Y2H assay

**Fig. 5:**
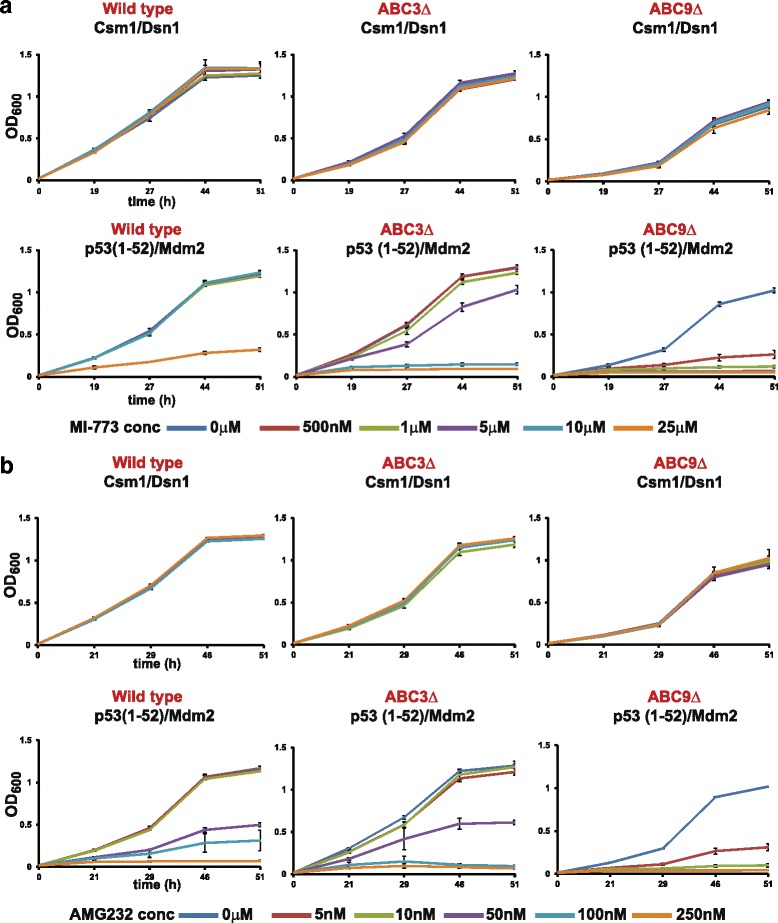
Inhibition of p53 (1–52)/Mdm2 interaction by AMG232 and MI-773 is more efficient in ABC9Δ cells in comparison to wild-type and ABC3Δ cells. **a** Overnight cultures of either wild-type, ABC3Δ cells, or ABC9Δ cells containing plasmids encoding either Gal4 AD-Csm1/Gal4 BD-Dsn1 or Gal4 AD-p53 (1–52)/Gal4 BD-Mdm2 in non-selective medium were washed in water and inoculated at OD_600_ = 0.2 into selective medium containing DMSO or MI-773 at the indicated concentrations in duplicate. For each strain, growth as measured by average OD_600_ of duplicate cultures is plotted at different time points following inoculation (0, 19, 27, 44, and 51 h). Ends of the vertical bar indicate the OD_600_ values of the duplicate cultures. **b** Same as a, but performed with AMG232

We then tested whether the Y2H assay for the p53 (1–52)/Mdm2 interaction optimized for nutlin was extendable to other p53-Mdm2 interaction inhibitors, namely AMG232 and MI-773. Like nutlin, AMG232 and MI-773 bind to the p53-binding site in Mdm2 and block the p53-Mdm2 interaction (Additional file 1: Figure S1). Both AMG232 and MI-773 abolished the p53 (1–52)/Mdm2 interaction in wild-type, ABC3Δ, and ABC9Δ strains (Fig. 5a, b). However, the p53 (1–52)/Mdm2 interaction was approximately 25-fold more sensitive to MI-773 and AMG232 in the ABC9Δ strain in comparison to the wild-type strain (Fig. 5a, b).

### The full-length p53/Mdm2 interaction is more resistant to small-molecule inhibitors in comparison to the p53 (1–52)/Mdm2 interaction

**Fig. 6:**
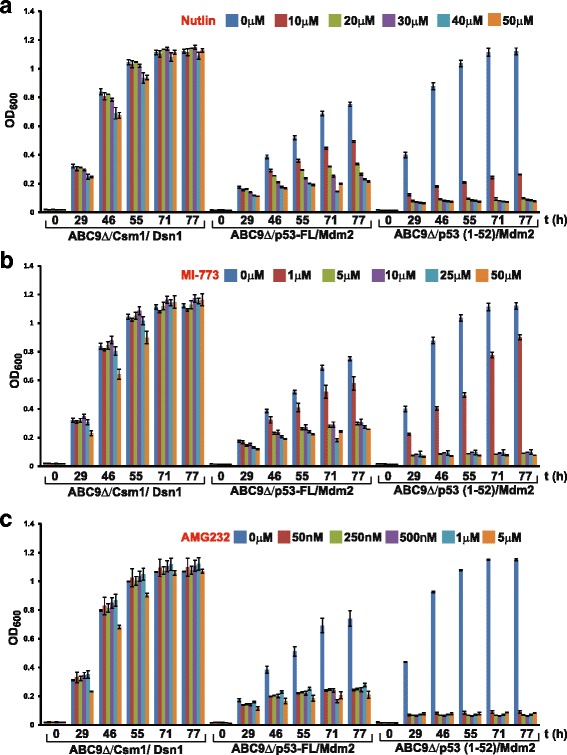
The full-length p53/Mdm2 interaction is more resistant to small-molecule inhibitors in comparison to the p53 (1–52)/Mdm2 interaction. **a** Overnight cultures of ABC9Δ cells containing plasmids encoding either Gal4 AD-Csm1/Gal4 BD-Dsn1, Gal4 AD-p53FL/Gal4 BD-Mdm2, or Gal4 AD-p53 (1–52)/Gal4 BD-Mdm2 in non-selective medium were washed in water and inoculated at OD_600_ = 0.2 into selective medium containing DMSO or nutlin at the indicated concentrations in duplicate. For each strain, growth as measured by average OD_600_ value of duplicate cultures is plotted at different time points following inoculation (0, 29, 46, 55, 71, and 77 h). Ends of the vertical bar indicate the OD_600_ values of the duplicate cultures. **b**, **c** Same as in a, but performed with MI-773 and AMG232, respectively

As the p53 (1–52)/Mdm2 interaction in the ABC9Δ strain was more sensitive to small-molecule inhibitors in comparison to wild-type and ABC3Δ strains (Fig. 5), we tested whether the full-length p53/Mdm2 interaction was sensitive to small-molecule inhibitors in the ABC9Δ strain. Nutlin, MI-773, and AMG232 inhibited the interaction of full-length p53 with Mdm2 by approximately 70–75% (Fig. 6). Inhibition of the p53-FL/Mdm2 interaction by nutlin in the ABC9Δ strain was approximately 2- to 3-fold better than that achieved in the ABC3Δ strain (Fig. 2a). In contrast, the p53 (1–52)/Mdm2 interaction was inhibited by approximately 90–95% by the three inhibitors (Fig. 6). Taken together, our results indicate that the interaction of full-length p53 with Mdm2 is more resistant to small-molecule inhibitors in comparison to the p53 (1–52)/Mdm2 interaction.

### Highly conserved Box II in the DNA-binding domain of p53 strengthens the p53-Mdm2 interaction

**Fig. 7:**
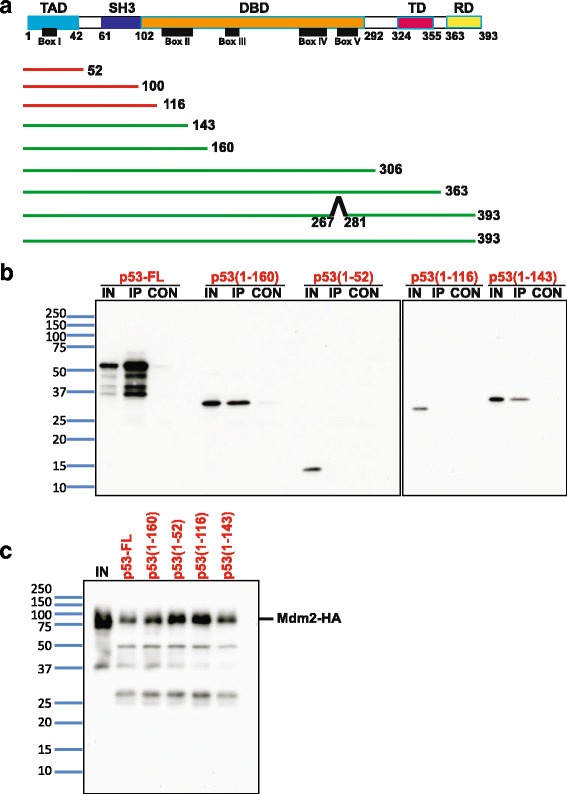
The Box II region of p53’s DNA-binding domain (DBD) promotes interaction with Mdm2. **a** Analyses of domains in p53 required for nutlin-resistant interaction with Mdm2 is summarized here. Locations of the transactivation domain (TAD), the proline rich SH3 domain, the DBD, the tetramerization domain (TD), the regulatory domain (RD), and the highly conserved Boxes (I to V) are indicated in the primary structure of human p53. The p53 constructs indicated by red and green lines are nutlin sensitive and nutlin resistant, respectively, in the Y2H assay with Mdm2. **b** Mdm2-HA, full-length p53 (p53-FL), p53 (1–160), p53 (1–52), p53 (1–116), and p53 (1–143) proteins were synthesized by in vitro translation. Binding assay was performed by incubating Mdm2-HA with the different p53 variants, followed by immunoprecipitation of Mdm2-HA using an anti-HA antibody and measuring the amount of co-immunoprecipitated p53 by western blotting analysis using anti-p53 (DO-1) antibody. Input (IN), immunoprecipitated proteins (IP), and eluate from control IP performed without Mdm2-HA (CON) were loaded on SDS-PAGE gels. **c** Amount of Mdm2-HA immunoprecipitated in b was analyzed by western blotting using an anti-HA antibody. IN is diluted five-fold in comparison to the IP samples for the different binding reactions (indicated in red)

To rationalize our observation that nutlin, AMG232, and MI-773 inhibited the p53 (1–52)/Mdm2 interaction much more efficiently than the full-length p53/Mdm2 interaction in the Y2H assay we hypothesized that there are additional domains in p53 besides the transactivation domain that interact with Mdm2. To map the region of p53 that contributes to nutlin resistance of the p53-Mdm2 interaction, we made a series of deletions of full-length p53 and assayed the nutlin sensitivity of their interaction with Mdm2. The results are summarized in Fig. 7a. Deletion of the C-terminal tail or the DNA-binding domain Box V of p53, which have been implicated in the interaction with Mdm2, had no effect on nutlin sensitivity. Interestingly, p53 (1–160), which has the first 60 residues of the DNA-binding domain, was resistant to nutlin and p53 (1–143) was partially resistant to nutlin. The interaction of p53 (1–160) with Mdm2 was abolished by the F19A mutation and by deletion of the transactivation domain (Additional file 8: Figure S8). These results indicate that, although the primary interaction of p53 (1–160) with Mdm2 is via the transactivation domain, the DNA-binding domain interacts with Mdm2 and stabilizes the interaction.

To map the domain of Mdm2 that is required for increased nutlin resistance of its interaction with full-length p53, we expressed the minimal Mdm2 (25–109) required for interaction with p53. The interaction of Mdm2 (25–109) with p53 (1–160) and p53 (full length) was also resistant to nutlin. To test whether the decreased sensitivity of p53-Mdm2 interaction was due to increased p53 levels, we compared the protein amounts of strains expressing different p53 variants by western blotting (Additional file 9: Figure S9). Full-length p53 was expressed to a lower extent in comparison to the other p53 variants, ruling out this possibility.

To directly compare the strength of interaction between Mdm2 and different variants of p53, we used an in vitro binding assay. We prepared HA-tagged Mdm2, full-length p53, p53 (1–52), and p53 (1–160) by in vitro translation. We then mixed HA-Mdm2 with the p53 variants and assayed the interaction by co-immunoprecipitation. Full-length p53 bound specifically to Mdm2 (Fig. 7b). Interestingly, p53 (1–160) bound to Mdm2 approximately 10-fold more efficiently than p53 (1–52). These results are consistent with our Y2H results, which show that the interaction of p53 (1–52) with Mdm2 is more sensitive to small-molecule inhibitors compared to the p53 (1–160)/full-length p53 and Mdm2 interactions. We also tested the ability of p53 (1–116) and p53 (1–143) to bind to Mdm2 in the in vitro binding assay. Only p53 (1–143), but not p53 (1–116), bound to Mdm2 (Fig. 7b). Comparable amounts of Mdm2-HA were immunoprecipitated in all the binding reactions (Fig. 7c). These results are in excellent agreement with our Y2H assay results.

**Fig. 8:**
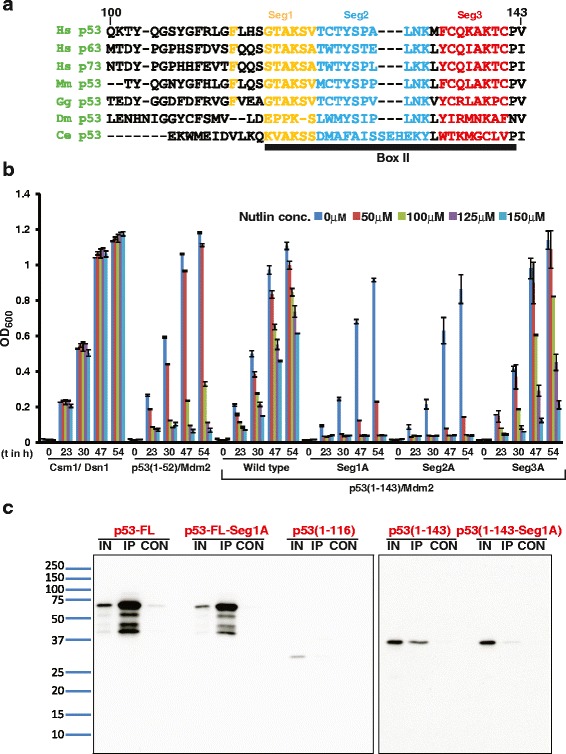
The conserved Segment 1 in p53’s DNA-binding domain promotes interaction with Mdm2. **a** Alignment of amino acid residues 100–143 of *Homo sapiens* p53 with the homologous amino acid sequences of *Homo sapiens* p63, *Homo sapiens* p73, *Mus musculus* p53, *Gallus gallus* p53, *Drosophila melanogaster* p53, and *Caenorhabditis elegans* p53. Conserved Segments 1, 2, and 3 are indicated in yellow, cyan, and red, respectively. Location of the conserved Box II is indicated by the black rectangle. **b** Overnight cultures of ABC3Δ cells containing plasmids encoding either Gal4 AD-Csm1/Gal4 BD-Dsn1, Gal4 AD-p53 (1–52)/Gal4 BD-Mdm2, Gal4 AD-p53 (1–143)/Gal4 BD-Mdm2, Gal4 AD-p53 (1–143-Seg1A)/Gal4 BD-Mdm2, Gal4 AD-p53 (1–143-Seg2A)/Gal4 BD-Mdm2, or Gal4 AD-p53 (1–143-Seg3A)/Gal4 BD-Mdm2 in non-selective medium were washed in water and inoculated at OD_600_ = 0.2 into selective medium containing DMSO or nutlin at the indicated concentrations. Growth of the cultures was monitored by recording the absorbance at 600 nm after 0, 23, 30, 47, and 54 h following inoculation. Ends of the vertical bar indicate the OD_600_ values of the duplicate cultures. **c** Mdm2-HA, full-length p53 (p53-FL), full-length p53 with Seg1A mutation (p53-FL-Seg1A), p53 (1–116), p53 (1–143), and p53 (1–143-Seg1A) were synthesized by in vitro translation. The binding assay was performed by incubating Mdm2-HA with the different p53 variants, followed by immunoprecipitation of Mdm2-HA using an anti-HA antibody and measuring the amount of co-immunoprecipitated p53 by western blotting analysis using anti-p53 (DO-1) antibody. Input (IN), immunoprecipitated proteins (IP), and eluate from control IP performed without Mdm2-HA (CON) were loaded on SDS-PAGE gels

To identify residues involved in the additional p53-Mdm2 interaction, we examined the conserved residues around the region (116–143) of p53 that is minimally required for nutlin resistance in the Y2H assay and stable interaction with Mdm2 in the in vitro binding assay (Fig. 8a). We split the conserved regions into three segments (Seg1–3). Interestingly, these segments lie very close to DNA in the p53-DNA co-crystal structure [34] (Additional file 10: Figure S10). We replaced the conserved residues in each segment with alanine. Mutations in segments 1 and 2, but not 3, rendered the p53 (1–143)/Mdm2 interaction nutlin sensitive (Fig. 8b). We further replaced the subset of residues in Segments 1 and 2 with alanine and determined their effect on the nutlin sensitivity of the p53 (1–143)/Mdm2 interaction. F113A (Seg1AA), G117A T118A (Seg1BA), and K120A S121A V122A (Seg1CA) mutations in Segment 1 conferred nutlin sensitivity (Additional file 11: Figure S11A). However, mutations T125A Y126A S127A (Seg2BA) and L130A N131A K132A (Seg2CA) in Segment 2 had no effect on nutlin resistance (Additional file 11: Figure S11A). The T123A C124A T125A Y126A S127A (Seg2AA) mutation in Segment 2 abolished growth in selective medium (Additional file 11: Figure S11A). We confirmed that the wild-type and mutant p53 (1–143) proteins were expressed at comparable levels by western blotting (Additional file 11: Figure S11B). As multiple mutations in Segment 1 conferred nutlin sensitivity, we focused on the Seg1 mutant for further analysis.

We tested whether the Seg1A mutant was defective in interaction with Mdm2 in the in vitro binding assay. Crucially, only wild-type p53 (1–143), but not its Seg1A mutant variant, interacted with Mdm2 in vitro (Fig. 8c). However, the Seg1A mutation did not have an effect on the full-length p53-Mdm2 interaction (Fig. 8c). Comparable amounts of Mdm2-HA were immunoprecipitated in all the binding reactions (Additional file 12: Figure S12). In agreement with this result, the Seg1A mutation also did not have any effect on the p53-FL/Mdm2 interaction in the Y2H assay (Additional file 13: Figure S13).

We suggest that additional domains in p53 that interact with Mdm2 mask the effect of Seg1A mutation on the p53-Mdm2 interaction. This is consistent with our observation that full-length p53 binds more strongly to Mdm2 in comparison to p53 (1–143) (Figs. 7b and 8c). Although the physiological significance of the Seg1/Mdm2 interaction remains to be clarified, there was an excellent correspondence between sensitivity of the p53-Mdm2 interaction to small-molecule inhibitors in the Y2H assay and their stable association in the in vitro binding assay.

### A two-phased strategy for screening of PPI inhibitors using the Y2H assay

**Fig. 9:**
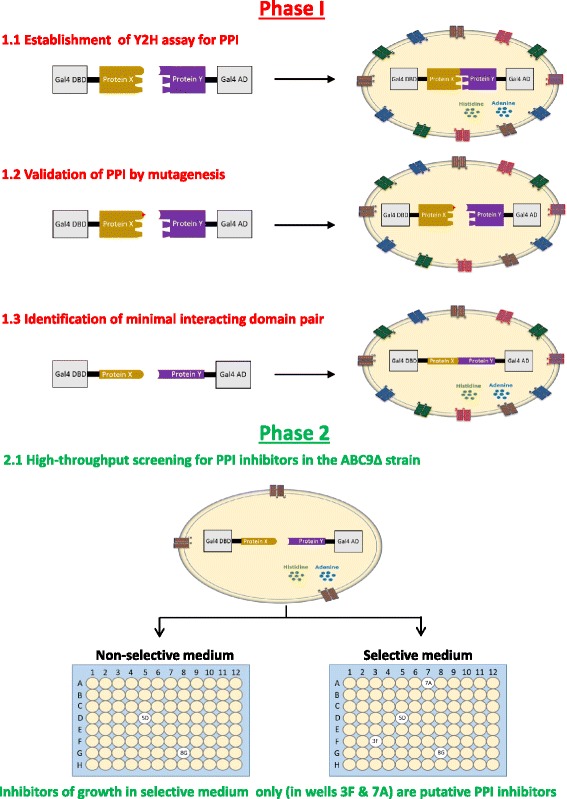
A two-phased strategy to screen for small-molecule inhibitors of PPI using the Y2H assay. Please see the text for details

The enhanced permeability of the ABC9Δ strain coupled with economic aspects of high-throughput, growth-based assays makes the Y2H assay suitable for screening of PPI inhibitors. In Fig. 9, we depict a methodological flowchart using a two-phased Y2H assay to screen for inhibitors of PPI. In the first phase, a Y2H assay for PPIs of interest is established and validated by mutagenesis. Based on our experience with the p53-Mdm2 interaction, it would be prudent to identify domains minimally required for PPIs by truncations as weak secondary PPIs affect the sensitivity to small-molecule inhibition. Experiments for the first phase can be performed in the standard wild-type yeast strain for two-hybrid analysis. In the second phase, the plasmids encoding the minimal set of specifically interacting protein domains are introduced into the ABC9Δ strain, which is permeable to small molecules. Transformants pre-grown in non-selective medium are split into two and transferred into selective and non-selective media containing small-molecule compounds in a microtiter plate. Compounds toxic to yeast cells will inhibit growth in both selective and non-selective media. However, compounds that inhibit the growth of yeast cells only in selective media are putative PPI inhibitors and can be taken up for further investigation.

## Discussion

By using the p53-Mdm2 interaction, which is amenable to small-molecule inhibition, as a model, we have developed and validated an improved version of the Y2H assay that can be used in a high-throughput manner. To accomplish this, we made two crucial changes. Firstly, we used the minimal Mdm2-interacting domain in p53 instead of full-length p53. Secondly, we increased the permeability of yeast cells to small molecules by disabling ABC transporters.

By analyzing the p53 domains required for conferring resistance to inhibition by small molecules, we identified a hitherto unreported Mdm2-interacting segment overlapping with the highly conserved Box II region in the core domain of p53. Mdm2 inhibits the DNA binding activity of p53, but precisely how this occurs remains unknown. Our observations offer an explanation for this. As the DNA domain of p53 is engaged in interactions with Mdm2, p53-Mdm2 complexes may have a restricted ability to bind DNA. It will be interesting to test the effect of mutating segment 1 in the core domain of p53 on the Mdm2 inhibition of the DNA-binding activity of p53 and on the function and stability of p53 in mammalian cells.

Why do small-molecule inhibitors inhibit the full-length p53-Mdm2 interaction in mammalian cells but not in the Y2H system? Since a good correlation between the strength of the p53-Mdm2 interaction in vitro and an ease of inhibition by small-molecule inhibitors in the Y2H assay was observed, we suggest that there are factors in mammalian cells that compete with Mdm2 in binding to p53. Displacement of the transcriptional activation domain of p53 from Mdm2 by small-molecule inhibitors might create a binding site for proteins like p300, which are not present in yeast cells. Alternatively, post-translational modifications in either p53 or Mdm2 in mammalian cells may weaken the p53-Mdm2 interaction. Our observations might explain why the p53-Mdm2 interaction is only partially inhibited by nutlin in vitro [35].

Complete inhibition of the p53 (1–52) interaction with Mdm2 by AMG232 and MI-773 at nanomolar concentrations suggests that the assay is highly sensitive. AMG232 was by far the most potent of the three inhibitors tested. This is consistent with its dissociation constant (0.045 nM) being lower than that of MI-773 (62 nM) and nutlin (90 nM). In contrast to nutlin, AMG232 and MI773 were able to inhibit the p53 (1–52)/Mdm2 interaction in wild-type cells. Nutlin thus appears to be a better substrate for yeast ABC transporters than MI-773 and AMG232. As many naturally occurring compounds, such as alkaloids, have an imidazole ring like nutlin, it is tempting to speculate that yeast ABC transporters have evolved to recognize such compounds. As human cells contain several ABC transporters, it will be interesting to test whether co-treatment of cancer cells with ABC transporter inhibitors improves the anti-cancer activity of nutlin.

We observed an excellent correlation between resistance of the p53-Mdm2 interaction to small-molecule inhibitors and the strength of the PPI in the in vitro binding assay. Binding of p53 (1–52/1–116) to Mdm2 is sensitive to nutlin in the Y2H assay and is too weak to be detected in the binding assay. In contrast, the binding of p53 (1–143/1–160) is resistant to nutlin in the Y2H assay and can be robustly detected in the binding assay. Binding of full-length p53 to Mdm2 was the strongest in the in vitro binding assay and resistant to nutlin in the Y2H assay. It is worth highlighting that the expression of p53 was approximately 10-fold lower compared to other p53 variants in yeast cells and its resistance to small-molecule inhibitors in the Y2H assay could be underestimated. Mutations in the Box II region of p53 (1–143) that render sensitivity to nutlin in the Y2H assay also abolish its association with Mdm2 in the in vitro binding assay. We therefore propose that the Y2H assay is not only useful in screening for PPI inhibitors but could also help to delineate domains involved in stabilizing PPIs.

## Conclusion

In summary, by disabling several ABC transporters in yeast, we developed a significantly improved Y2H assay to screen for small-molecule inhibitors of PPIs. Using inhibitors of the p53-Mdm2 interaction to validate the Y2H assay and demonstrate its suitability for high-throughput screening, we have also identified a novel putative Mdm2-binding site in the highly conserved Box II region of p53. The ease and economic nature of the assay, coupled with advantages of an in vivo-based system, makes it a valuable addition to the existing stockpile of in vitro tools to screen for PPI inhibitors.

## Methods

### Plasmid construction

The human p53 and Mdm2 ORF’s were PCR amplified using Phusion polymerase (NEB, Ipswich, MA, USA) from plasmids encoding p53 cDNA and Mdm2 cDNA and cloned into pGADT7-AD and pGBKT7-BD, respectively, by gap-repair [36]. Plasmids were isolated from yeast transformants, rescued in *E. coli* and their integrity was confirmed by sequencing. Truncated p53 (1–52, 1–100, 1–116, 1–143, 1–160, 1–306, 1–363, 43–393) and Mdm2 (25–109, 1–125), and mutant p53 (F19A, Seg mutants and Box-V deletion (Δ267–281) and Mdm2 (M62A) were generated using a similar approach. Plasmids streptavidin-binding peptide (SBP)-p53-pET22b (+) and HA-Mdm2-pET22b (+) were gifts from Dr Farid Ghadessy (p53 laboratory, Singapore). A list of plasmids used in the study is available in Additional file 14: Table S1.

### Yeast strain construction

Derivatives of the yeast strain AH109 in the MATCHMAKER GAL4 Two-Hybrid system (Clontech Laboratories, CA, USA) were used in this study. ABC transporter-related genes were deleted by standard methods using PCR product-mediated homologous recombination. The ABC9Δ strain was generated by crossing single deletion strains with each other. A list of yeast strains used in the study is available in Additional file 15: Table S2.

### Drug sensitivity test using the Y2H assay

(±)-Nutlin-3 (Cayman Chemicals, Ann Arbor, MI, USA) was dissolved in DMSO to make 50 mM stock solution. MI-773 (MedChem Express Co. Ltd., China) and AMG232 (MedChem Express) were dissolved in DMSO to make 10 mM stock solutions. Overnight yeast cultures were washed and then diluted to OD_600_ = 0.2 in 1 mL SD-Leu-Trp-His-Ade medium containing the desired concentration of the drug. The cultures were then placed in a 30 °C incubator with shaking for 3 days. Growth of the cultures was monitored by measuring the absorbance at 600 nm using the Implen Nanophotometer. This assay was also performed in a 96-well plate format with 200 μL SD-Leu-Trp-His-Ade medium containing the desired concentration of the drug in duplicate. OD_600_ at different time points was measured using a Gen 5^TM^ (BIO-TEK Instrument, Vermont, USA) microplate reader. The average of the two OD_600_ readings was calculated and used in the analysis.

### In vitro transcription–translation

The SBP-p53 and HA-Mdm2 ORF’s were amplified using SBP-p53-pET22b (+) and HA-Mdm2-pET22b (+) vectors as templates. Truncated (1–52, 1–116, 1–143, and 1–160) and mutated p53 were generated by overlap PCR. These PCR products were used as substrates for in vitro translation (IVT) using PURExpress in vitro protein synthesis kit according to the instruction manual (NEB, Ipswich, MA, USA).

### Immunoprecipitation

IVT-expressed wild-type, truncated, or mutant p53 were pre-cleared with Protein G beads (Invitrogen, Carlsbad, CA, USA), followed by incubation with IVT-expressed Mdm2 at room temperature for 1 h. Anti-HA 3 F10 antibody (Roche Life Science, USA) at 1:200 dilution was added into the p53/Mdm2 mixture and incubated at 4 °C for 1 h. Protein G beads were pre-blocked with 3% BSA/PBS and then added into the p53-Mdm2-anti-HA 3 F10 mixture. The beads were then washed once in 1× lysis buffer (50 mM Tris-HCl pH 8.0, 5 mM EDTA pH 7.4, 150 mM NaCl, 0.5% NP-40, and 1 mM DTT), followed by two washes with 1× SNNTE buffer (50 mM Tris-HCl pH 7.4, 5 mM EDTA pH 7.4, 5% sucrose, 1% NP-40, 500 mM NaCl, and 1 mM DTT) and finally washed with 1× lysis buffer. Bound proteins were eluted by adding 40 μL SDS-PAGE loading buffer to the tubes followed by incubation at 95 °C for 5 minutes.

### Western blot analysis

Protein samples were resolved by electrophoresis on 12% SDS-PAGE gels and transferred onto nitrocellulose membranes. Blots were blocked with 5% milk in PBS/0.1% Tween 20. For p53, blots were probed with anti-p53 DO-1 antibody conjugated with horseradish peroxidase (1:1000; Santa Cruz Biotechnology, Santa Cruz, CA, USA). For Mdm2, the blot was probed with anti-HA 3F10 antibody (1:2000; Roche Life Science, USA) followed by goat anti-rat HRP-conjugated antibody (1:5000; Santa Cruz Biotechnology). Blots were developed with ECL prime western blotting detection reagent (Amersham Pharmacia Biotech, USA).

## Additional files

Additional file 1: Figure S1. Crystal structures of Mdm2 bound to p53 transactivation domain (17–29), nutlin, MI-773, and AMG232. (A) Mdm2 in magenta surface bound to the TAD1 of p53 (residues 17–29) in yellow ribbon with its three critical hydrophobic residues F19, W23, and L26 shown in sticks (PDB id: 1YCR) [17]. (B, C, D) Mdm2 in magenta surface bound to nutlin-3A (PDB id: 1RV1) [19], MI-773 (PDB id: 5TRF) [20], and AMG232 (PDB id: 4WT2) [21], respectively, in sticks. Images were generated using PyMOL (DeLano, W. L. The PyMOL Molecular Graphics System, DeLano Scientific, 2002). (EPS 6007 kb)

Additional file 2: Figure S2. Nutlin inhibits the p53 (1–52)/Mdm2 interaction in ABC3Δ cells. (A) Overnight cultures of AH109 yeast cells containing plasmids encoding either Gal4 AD-p53/Gal4 BD-Mdm2 in non-selective medium were washed in water and inoculated into selective medium at OD_600_ = 0.2 with nutlin at the indicated concentrations. Growth of the cultures was monitored by recording the absorbance at 600 nm at 0, 32, and 49 h following inoculation. (B) Overnight cultures of ABC3Δ cells containing plasmids encoding either Gal4 AD-Csm1/Gal4 BD-Dsn1 or Gal4 AD-p53/Gal4 BD-Mdm2 in non-selective medium were washed in water and inoculated at OD_600_ = 0.2 into selective and non-selective medium containing DMSO or nutlin at the indicated concentrations. Growth of the cultures was monitored by recording the absorbance at 600 nm at the indicated time points. (C) Overnight cultures of ABC3Δ cells containing plasmids encoding either Gal4 AD-Csm1/Gal4 BD-Dsn1 or Gal4 AD-p53 (1–52)/Gal4 BD-Mdm2 in non-selective medium were washed in water and inoculated at OD_600_ = 0.2 into non-selective and selective medium containing DMSO or nutlin at the indicated concentrations. Growth of the cultures was monitored by recording the absorbance at 600 nm at the indicated time points. (EPS 1597 kb)

Additional file 3: Figure S3. M62A mutation in Mdm2 abolishes the nutlin sensitivity of the p53 (1–52)/Mdm2 (1–125) interaction. Overnight cultures of ABC3Δ cells containing plasmids encoding either Gal4 AD-Csm1/Gal4 BD-Dsn1 or Gal4 AD-p53 (1–52)/Gal4 BD-Mdm2 or Gal4 AD-p53 (1–52)/Gal4 BD-Mdm2 (1–125) or Gal4 AD-p53 (1–52)/Gal4 BD-Mdm2 (1–125-M62A) in non-selective medium were washed in water and inoculated at OD_600_ = 0.2 into selective medium containing DMSO or nutlin at the indicated concentrations. Growth of the cultures was monitored by recording the absorbance at 600 nm at the indicated time points. (EPS 1261 kb)

Additional file 4: Figure S4. Nutlin is a substrate of the yeast ABC transporter Pdr5. Overnight cultures of either wild-type or *pdr5Δ* or *pdr1Δ* or *pdr3Δ* or *pdr5Δ pdr1Δ pdr3Δ* cells containing plasmids encoding Gal4 AD-p53 (1–52)/Gal4 BD-Mdm2 were washed in water and inoculated at OD_600_ = 0.2 into selective medium containing DMSO or nutlin at the indicated concentrations. Growth of the cultures was monitored by recording the absorbance at 600 nm after 0, 23, 46, and 71 h following inoculation. (EPS 1323 kb)

Additional file 5: Figure S5. ABC transporters have distinct substrate specificities. (A) Overnight cultures of either wild-type or the different deletion strains, containing plasmids encoding Gal4 AD-p53 (1–52)/Gal4 BD-Mdm2, were washed in water and inoculated at OD_600_ = 0.2 into selective medium containing DMSO or nutlin at the indicated concentrations. Growth of the cultures was monitored by recording the absorbance at 600 nm after 0 (1), 24 (2), 44 (3), and 68 h (4) following inoculation. (B) Similar to A, but with rapamycin at the indicated concentrations. Absorbance measurements were performed after 0 (1), 21 (2), and 29 h (3). (EPS 1629 kb)

Additional file 6: Figure S6. ABC transporters have distinct substrate specificities. Repeat of the experiment described in Additional file 5: Figure S5A. (EPS 1316 kb)

Additional file 7: Figure S7. Nutlin sensitivity of p53 (1–52)/Mdm2 interaction is enhanced in the ABC9Δ strain in comparison to ABC3Δ and wild-type strains. Overnight cultures of ABC3Δ or ABC9Δ cells containing plasmids encoding either Gal4 AD-Csm1/Gal4 BD-Dsn1 or Gal4 AD-p53 (1–52)/Gal4 BD-Mdm2 in non-selective medium were washed in water and inoculated into selective medium at OD_600_ = 0.2. Growth of the cultures was monitored by recording the absorbance at 600 nm after 0, 30, 48, and 72 h following inoculation. (EPS 1329 kb)

Additional file 8: Figure S8. The transactivation domain is required for the interaction of p53 (1–160) with Mdm2. Overnight cultures of AH109 yeast cells containing plasmids encoding either Gal4 AD-p53 (1–160)/Gal4 BD-Mdm2 or Gal4 AD-p53 (1–160-F19A)/Gal4 BD-Mdm2 or Gal4 AD-p53 (43–160)/Gal4 BD-Mdm2 in non-selective medium were washed in water and inoculated into selective and non-selective medium at OD_600_ = 0.2 in triplicate. Growth of the cultures was monitored by recording the absorbance at 600 nm after 0 (1), 19 (2), 28 (3), 44 (4), 51 (5), and 65 h (6) following inoculation. Average absorbance of the three cultures at different time points is indicated along with the error bars. (EPS 1397 kb)

Additional file 9: Figure S9. The Gal4 AD-full-length p53 fusion is expressed poorly compared to the other p53 variants. Protein extracts from logarithmically growing ABC9Δ cells containing plasmids encoding the indicated Gal4 AD- and Gal4 BD-fusion proteins were electrophoresed by SDS-PAGE and the p53 and Mdm2 proteins were detected by western blotting using anti-HA and anti-myc antibodies. Cdc28 served as a loading control. (EPS 24410 kb)

Additional file 10: Figure S10. Location of residues in Segments 1, 2, and 3 in the structure of p53’s DNA-binding domain (DBD) complexed to DNA. p53’s DBD (residues 95–293) is shown in green in the cartoon with the zinc atom shown as a cyan sphere. DNA (GGACATGTCCG) is shown in orange in the cartoon with blue sticks representing the bases. Segment 1 (F113 + G117-V122), Segment 2 (T123-A129), and Segment 3 (F134-V143) are shown in yellow, violet, and magenta, respectively. Image was generated using PyMOL (DeLano, W. L. The PyMOL Molecular Graphics System, DeLano Scientific, 2002) with the crystal structure of p53-DNA complex (PDB id: 2AHI) [34]. (EPS 1572 kb)

Additional file 11: Figure S11. Mutations in Segment 1 in p53’s DNA-binding domain confer nutlin sensitivity to the p53 (1–143)/Mdm2 interaction. Overnight cultures of ABC3Δ cells containing plasmids encoding either Gal4 AD-Csm1/Gal4 BD-Dsn1 or Gal4 AD-p53 (1–52)/Gal4 BD-Mdm2 or Gal4 AD-p53 (1–143)/Gal4 BD-Mdm2 or Gal4 AD-p53 (1–143-Seg1AA)/Gal4 BD-Mdm2 or Gal4 AD-p53 (1–143-Seg1BA)/Gal4 BD-Mdm2 or Gal4 AD-p53 (1–143-Seg1CA)/Gal4 BD-Mdm2 or p53 (1–143-Seg2AA)/Gal4 BD-Mdm2 or Gal4 AD-p53 (1–143-Seg2BA)/Gal4 BD-Mdm2 or Gal4 AD-p53 (1–143-Seg2CA)/Gal4 BD-Mdm2 in non-selective medium were washed in water and inoculated at OD_600_ = 0.2 into selective medium containing DMSO or nutlin at the indicated concentrations. Growth of the cultures was monitored by recording the absorbance at 600 nm after 0 (1), 20 (2), 27 (3), 44 (4), and 51 h (5) following inoculation. Residues replaced with alanine in the various mutants are indicated in red. Ends of the vertical bar indicate the OD_600_ values of the duplicate cultures. (A) Protein extracts from logarithmically growing ABC9Δ cells containing plasmids encoding the indicated Gal4 AD- and Gal4 BD-fusion proteins were electrophoresed by SDS-PAGE and the p53 and Mdm2 proteins were detected by western blotting analysis using anti-HA and anti-myc antibodies, respectively. Beta-actin served as a loading control. (EPS 19340 kb)

Additional file 12: Figure S12. The conserved Segment 1 in p53’s DNA-binding domain promotes interaction with Mdm2. Amounts of Mdm2-HA immunoprecipitated in the samples from the binding reactions described in Fig. 8c were analyzed by western blotting using anti-HA antibody. (EPS 2200 kb)

Additional file 13: Figure S13. Mutations in Segment 1 in p53’s DNA-binding domain does not affect nutlin sensitivity of the p53-FL/Mdm2 interaction. Overnight cultures of ABC3∆ cells containing plasmids encoding either Gal4 AD-Csm1/Gal4 BD-Dsn1 or Gal4 AD-p53 (1–52)/Gal4 BD-Mdm2 or Gal4 AD-p53/Gal4 BD-Mdm2 or Gal4 AD-p53-Seg1A/Gal4 BD-Mdm2 in non-selective medium were washed in water and inoculated at OD_600_ = 0.2 into selective medium containing DMSO or nutlin at the indicated concentrations. Growth of the cultures was monitored by recording the absorbance at 600 nm after 0 (1), 23 (2), 30 (3), 47 (4), and 54 h (5) following inoculation. Ends of the vertical bar indicate the OD_600_ values of the duplicate cultures. (EPS 1438 kb)

Additional file 14: Table S1. List of plasmids used in the study. (PDF 66 kb)

Additional file 15: Table S2. List of yeast strains used in the study. (PDF 111 kb)

## Abbreviations

PPI: protein–protein interaction
SBP: streptavidin-binding peptide
SD: synthetic dropout
Y2H: yeast two-hybrid assay
