# Supplementary material for: A yeast two-hybrid system for the screening and characterization of small-molecule inhibitors of protein–protein interactions identifies a novel putative Mdm2-binding site in p53
Source: BMC Biol. 2017 Nov 9;15:108. doi: 10.1186/s12915-017-0446-7 (PMC5680816; doi:10.1186/s12915-017-0446-7)
Supplement: Supplementary file 14 — List of plasmids used in the study. (PDF 66 kb) [file 12915_2017_446_MOESM14_ESM.pdf]

Additional file 14: Table S1

**Plasmid No.**	**Plasmid Name**	**Used in Figure no.**
674	pGADT7 AD	1
675	pGBKT7	1
667	pGADT7 AD- p53	1, 2, 6, S2, S9, S13
668	pGBKT7-Mdm2	1-8, S2-S9, S11, S13
830	pGADT7 AD- p53 (F19A)	1
831	pGADT7 AD- p53 (43-393)	1
667	pGADT7 AD- p53 (1-52)	2-8, S2-S9, S11, S13
694	pGBKT7-Mdm2 (1-125)	2, S3
753	pGBKT7-Mdm2 (1-125) (M62A)	2, S3
793	pADH1-*PDR5* -2µ Ori-URA3	3
755	pGADT7 AD- p53 (1-100)	7, S9
756	pGADT7 AD- p53 (1-116)	7
757	pGADT7 AD- p53 (1-143)	7, 8, S9, S11
670	pGADT7 AD- p53 (1-160)	7, S8, S9
762	pGADT7 AD- p53 (1-306)	7, S9
764	pGADT7 AD- p53 (1-363)	7
763	pGADT7 AD- p53 (Δ 267-281)	7
800	pET22b (+)-SBP- p53	7
799	pET22b (+)-Mdm2 HA	7
795	pGADT7-p53 (1-143) Seg 1A- **A**LHS**AA**A**AA**	8
796	pGADT7-p53 (1-143) Seg 2A- **AAAAA**PA**AAA**	8
797	pGADT7-p53 (1-143) Seg 3A- **AAAA**AA**AA**	8
758	pGADT7 AD- p53(1-160) (F19A)	S8
759	pGADT7 AD- p53(43-160)	S8
760	pGBKT7-Mdm2 (25-109)	S9
801	pGADT7-p53 (1-143) Seg 1AA- **A**LHSGTAKSV	S11
802	pGADT7-p53 (1-143) Seg 1BA - FLHS**AA**AKSV	S11
803	pGADT7-p53 (1-143) Seg 1CA- FLHSGTA**AAA**	S11
804	pGADT7-p53 (1-143) Seg 2AA- **AAAAA**PALNK	S11
805	pGADT7-p53 (1-143) Seg 2BA- TC**AAA**PALNK	S11
806	pGADT7-p53 (1-143) Seq 2CA- TCTYSPA**AAA**	S11
823	pGADT7-p53 Seg 1A- **A**LHS**AA**A**AA**	S13
