# Supplementary material for: A yeast two-hybrid system for the screening and characterization of small-molecule inhibitors of protein–protein interactions identifies a novel putative Mdm2-binding site in p53
Source: BMC Biol. 2017 Nov 9;15:108. doi: 10.1186/s12915-017-0446-7 (PMC5680816; doi:10.1186/s12915-017-0446-7)
Supplement: Supplementary file 15 — List of yeast strains used in the study. (PDF 111 kb) [file 12915_2017_446_MOESM15_ESM.pdf]

Additional file 15: Supplementary Table S2

**Yeast strain**	**Genotype**
AH109	*MATa trp1-901 leu2-3,112 ura3-52 his3-200 gal4Δ gal80Δ LYS2 : : GAL1UAS–GAL1TATA–HIS3 GAL2UAS–GAL2TATA–ADE2 URA3 : : MEL1UAS–MEL1TATA AUR1-C MEL1*
ABC3Δ	*MAT α pdr1:KanMX6 pdr3:KanMX6 pdr5: KanMX6* (otherwise same as AH109)
ABC9Δ	*MAT α pdr1:KanMX6 pdr3:KanMX6 pdr5: KanMX6 snq2:HphMX6 pdr10:HphMX6 pdr11: HphMX6 pdr15:NatMX6 yor1:NatMX6 ycf1:NatMX6* (otherwise same as AH109)
3459	*pdr1:KanMX6* (otherwise same as AH109)
3461	*pdr3:KanMX6* (otherwise same as AH109)
3463	*pdr5: KanMX6* (otherwise same as AH109)
3586	*snq2:HphMX6* (otherwise same as AH109)
3587	*pdr10:HphMX6* (otherwise same as AH109)
3589	*pdr11: HphMX6* (otherwise same as AH109)
3590	*pdr15:NatMX6* (otherwise same as AH109)
3592	*yor1:NatMX6* (otherwise same as AH109)
3594	*ycf1:NatMX6* (otherwise same as AH109)
